# Smoking Cessation Treatment for Parents Who Dual Use E-Cigarettes and Traditional Cigarettes

**DOI:** 10.1155/2021/6639731

**Published:** 2021-03-17

**Authors:** Emara Nabi-Burza, Jeremy E. Drehmer, Bethany Hipple Walters, M. C. Willemsen, Maurice P. A. Zeegers, Jonathan P. Winickoff

**Affiliations:** ^1^Massachusetts General Hospital for Children, Division of General Academic Pediatrics, Boston, MA, USA; ^2^Massachusetts General Hospital, Tobacco Research and Treatment Center, Boston, MA, USA; ^3^Department of Health Promotion, Maastricht University, Maastricht, Netherlands; ^4^Dutch Alliance for a Smokefree Society, The Hague, NL, Netherlands; ^5^Nutrition and Translational Research in Metabolism (School NUTRIM), Maastricht University, Maastricht, Netherlands; ^6^Care and Public Health Research Institute (School CAPHRI), Maastricht University, Maastricht, Netherlands; ^7^Harvard Medical School, Boston, MA, USA; ^8^American Academy of Pediatrics, Julius B. Richmond Center of Excellence, Itasca, IL, USA

## Abstract

**Introduction:**

An increasing number of parents use both e-cigarettes and cigarettes (dual users). Previous studies have shown that dual users may have higher rates of contemplating smoking cessation than parents who only smoke cigarettes. This study was aimed to assess the delivery of tobacco cessation treatment (prescription for nicotine replacement therapy and referral to the quitline) among parents who report being dual users vs. cigarette-only smokers.

**Methods:**

A secondary analysis of parent survey data collected between April and October 2017 at 10 pediatric primary care practices participating in a cluster-randomized controlled trial of the Clinical Effort Against Secondhand Smoke Exposure (CEASE) intervention was conducted. Parents were considered to be dual users of cigarettes and e-cigarettes if they reported smoking a cigarette, even a puff, in the past seven days and using an e-cigarette within the past 30 days. Parents were asked if they received a prescription for nicotine replacement therapy and referral to the quitline to help them quit from their child's clinician. Multivariable logistic regression examined factors (dual use, insurance status, relationship to the child, race, and education status of the parent) associated with delivery of smoking cessation treatment (receiving prescriptions and/or enrollment in quitline) to smoking parents. Further, we compared the rates of tobacco cessation treatment delivery to dual users in the usual-care control practices vs. intervention practices.

**Results:**

Of 1007 smokers or recent quitters surveyed in the five intervention practices, 722 parents reported current use of cigarettes-only and 111 used e-cigarettes. Of these 111 parents, 82 (73.9%) reported smoking cigarettes. Parents were more likely to report receiving any treatment if they were dual users vs. cigarette-only smokers (OR 2.43, 95% CI 1.38, 4.29). Child's insurance status, parents' sex, education, and race were not associated with parental receipt of tobacco cessation treatment in the model. No dual users in the usual-care control practices reported receiving treatment. *Discussion*. Dual users who visited CEASE intervention practices were more likely to receive treatment than cigarette-only smokers when treatments were discussed. An increased uptake of tobacco cessation treatments among dual users reinforces the importance of discussing treatment options with this group, while also recognizing that cigarette-only smokers may require additional intervention to increase the acceptance rate of cessation assistance. This trial is registered with ClinicalTrials.gov, Identifier: NCT01882348.

## 1. Background

Parental smoking exposes children to thousands of harmful chemicals and toxins in tobacco smoke, increasing the risk of ear infections and respiratory infections such as bronchiolitis and pneumonia in infants and young children [1]. In addition to the increased risk of infections, exposure to tobacco smoke is associated with an increased risk of learning problems and attention-deficit/hyperactivity disorder (ADHD) in children [2]. Despite the strong evidence of harm to children from exposure to tobacco smoke, almost 500 million children worldwide are exposed to secondhand smoke at home [3]. According to National Health and Nutrition Examination Survey (NHANES) data from 2013 to 2016, 38.1% of children aged 3–11 years were exposed to tobacco smoke in the United States (U.S.) [4].

There is no safe level of tobacco smoke exposure, and the only way to prevent children from this exposure is for parents to quit smoking [1]. According to the 2008 update of the US Public Health Service Guideline for the Treatment of Tobacco Use and Dependence [5], behavioral counseling and cessation medications like nicotine replacement therapy (NRT) are each more effective than placebo or no treatment and even more effective when used in combination. NRT is the preferred pharmacological treatment that should be offered to parents by a pediatric healthcare provider as it is available over the counter, has an excellent safety profile, and is effective in achieving abstinence [6–8]. Nicotine is the main addictive substance in tobacco, but each puff of a cigarette also contains a mixture of thousands of compounds, including more than 60 well-established carcinogens [1, 9]. NRT helps to increase abstinence by replacing the nicotine—in the form of gum, patches, sprays, inhalers, or lozenges—while not containing the other harmful chemicals in tobacco smoke [8]. With a written prescription from a licensed practitioner, NRT is also covered by most insurance plans in the United States, including Medicaid. The evidence also suggests that proactive quitline counseling, when provided alone or in combination with cessation medications, increases rates of smoking cessation [10, 11].

Electronic cigarettes (e-cigarettes) are increasing in popularity, and their use is rapidly growing. They are perceived by some as a less toxic alternative to traditional cigarettes or used as a smoking cessation aid [12]. The U.S. Preventive Services Task Force [13], the latest U.S. Surgeon General's report on smoking cessation [11], a 2016 Cochrane review on the use of electronic cigarettes for smoking cessation [14], and the latest Public Health England report [15] have all concluded that the current evidence is insufficient to recommend e-cigarettes for smoking cessation. Despite inconclusive evidence to support e-cigarettes as cessation products, many smokers who are trying to quit smoking use them [16, 17]. Many smokers who use e-cigarettes have reportedly continued to smoke cigarettes (hereafter referred as dual users) [18]. Data from the 2016 Behavioral Risk Factor Surveillance System survey estimated that 54.6 percent of current e-cigarette users were also current smokers [19].

Parents who use e-cigarettes and continue to smoke cigarettes may be exposing their children to the harmful chemicals present in both tobacco smoke and e-cigarette vapor [20, 21]. E-cigarette aerosol contains nicotine and other potentially harmful chemicals including ultrafine particles that can be inhaled deep into the lungs, volatile organic compounds, and heavy metals, such as nickel, tin, and lead [22]. Nicotine exposure is known to harm the developing brains of children and adolescents [23, 24]. E-liquid for e-cigarettes usually contains a high concentration of nicotine which can be toxic if absorbed through the skin or ingested, posing a poisoning risk to children [25, 26]. Despite these known harmful effects of using e-cigarettes around children, one study showed that parents who used e-cigarettes were unaware of the potential health and safety hazards associated with their use and storage [27].

Research has shown that higher proportions of dual users have the strong intention to quit compared to cigarette smokers [28]. A 2019 paper showed that parents who use both e-cigarettes and cigarettes may have higher rates of contemplating smoking cessation than parents who only smoke cigarettes, suggesting that these parents may be using e-cigarettes for harm reduction or as a step towards cessation [29]. Since some dual users seem to have taken their first step towards smoking cessation by initiating e-cigarettes, there may be an opportunity to facilitate smoking and e-cigarette cessation using evidence-based treatments.

Child healthcare practices are ideal settings to identify parents who smoke and or use e-cigarettes and offer them evidence-based cessation treatments [30]. No previous studies have assessed the receipt of evidence-based smoking cessation treatments among this motivated group of parental dual users in the child healthcare setting. Therefore, this study was aimed to assess the receipt of evidence-based tobacco cessation treatments among parents who use both cigarettes and e-cigarettes compared to cigarettes only by their child's healthcare provider in the pediatric practices that delivered the Clinical Effort Against Secondhand Smoke Exposure (CEASE) intervention. Further, we compared the rates of tobacco cessation treatment delivery to dual users in the usual-care control practices vs. intervention practices.

## 2. Methods

A secondary analysis of data collected from ten pediatric practices in five U.S. states (TN, IN, VA, NC, and OH) randomized to the usual-care control and intervention arms of the CEASE trial was conducted between September and December 2019 [31]. The CEASE intervention consists of training child healthcare providers to routinely screen for parental tobacco use and offer evidence-based assistance to parents who smoke (enrollment in the state tobacco quitline, prescribing NRT, and advising families to establish smoke-free homes and cars) [31]. Parental exit interviews were conducted between April and October 2017, two years after the implementation of the intervention.

### 2.1. Study Sample

Research assistants conducted interviews with parents after their child's visit to the pediatric office between April and October 2017. Parents were eligible to enroll in the study and answer a detailed survey about their tobacco use and smoking characteristics if they reported smoking at least 100 cigarettes in their lifetime and if they had smoked a cigarette, even a puff in the last 7 days or had quit smoking within the past 2 years. Exit interviews continued until approximately 200 eligible parents completed the detailed survey at each practice, resulting in a study sample of 1,007 parents in the intervention practices and 943 in the control practices.

### 2.2. Measures

Parents were considered to be dual users of cigarettes and e-cigarettes if they reported smoking a cigarette, even a puff, in the past seven days and using an e-cigarette within the past 30 days. The primary outcome, as assessed by parental self-report at the exit interview, was the rate of receipt of tobacco cessation treatments among dual users vs. cigarette-only smokers. Parents were asked the following questions about receipt of tobacco cessation treatments as part of the detailed survey:

During your visit today, did a doctor, nurse, or other healthcare provider:

1. Ask if you smoke cigarettes
2. Advise you to quit smoking or stay quit
3. Discuss medicine to help you quit smoking or stay quit
4. Give you a prescription for medicine to help you quit smoking or stay quit
5. Discuss using a telephone “quitline” or other program
6. Enroll you in a telephone “quitline” or other program

Rates of parents asked about smoking, advised to quit smoking, advised about how the free tobacco control quitline could help them quit smoking, assisted with smoking cessation by discussing medications to help quit smoking and offering a prescription for NRT, and referral to the quitline were compared between dual users vs. cigarette-only smokers, as well as between intervention practices and usual-care control practices. Parents were considered to have received tobacco cessation treatment if they answered “yes” to receiving a prescription for medicine to help them quit and/or being enrolled in the telephone quitline. Rates of delivering tobacco cessation assistance were compared between dual users vs. cigarette-only smokers and, also, between intervention practices and usual-care control practices. Parents were considered to have received tobacco cessation treatment if they answered “yes” to receiving a prescription for medicine to help them quit and/or being enrolled in the telephone quitline. Rates of delivering tobacco cessation assistance were compared between dual users vs. cigarette-only smokers and, also, between intervention practices and usual-care control practices.

### 2.3. Statistical Analysis

Bivariate analyses were conducted using chi-square tests to explore the association between parent and child characteristics for parents who visited an intervention arm practice who received tobacco cessation treatment vs. those who did not receive any treatment. This analysis was limited to practices randomized to the intervention arm of the trial since assistance delivery was almost negligible in the control arm. Variables that had theoretical plausibility were added step-wise to a logistic regression model. Odds ratios (OR) and 95% confidence intervals (CI) were reported for each variable from the final model. All *p* values are two-sided and were considered significant at *p* < 0.05. Analyses were conducted using the Stata statistical software (StataCorp, 2017. Stata Statistical Software: Release 15. College Station, TX: Stata Corporation).

## 3. Results

Of the 1007 parents who completed the detailed survey after their child's visit in the intervention practices, 804 (79.8%) were current cigarette smokers (including 722 parents who reported current use of cigarettes only) and 203 (20.2%) were recent quitters. Overall, 111 parents (28.3%) reported current use of e-cigarettes, and of these, 82 parents (73.9%) also reported using cigarettes. These 82 parents met our definition of dual use.

Of the 943 parents who completed the detailed survey after their child's visit in the control practices, 727 (77.1%) were current smokers (including 646 parents who reported current use of cigarettes only) and 216 (22.9%) were recent quitters. Overall, 115 parents (12.2%) reported current use of e-cigarettes, and of these, 81 parents (70.4%) also reported using cigarettes. These 81 parents met our definition of dual use.

In the intervention practices, of the total 804 parents who were current smokers and answered questions about discussing and receiving treatment at that day's visit, 113 (14.1%) reported receiving tobacco cessation treatment at that day's visit.  Table 1 shows that 50% of parents who received tobacco cessation treatment were high school graduates compared to 43% who did not receive any treatment. Almost 88% of the parents who received treatment were planning to quit in the next 6 months compared to 75.4% who did not receive treatment. Similarly, almost 80% of the parents who received treatment were planning to quit in the next 30 days compared to 64% parents who did not receive any treatment.

The overall rates of screening parents for tobacco use, discussing using NRT, and discussing referral to the quitline and receipt of tobacco cessation treatments (NRT prescription and quitline referral) were higher among parents who were dual users of e-cigarettes and cigarettes compared to cigarette-only smokers in the intervention arm (Table 2). Of the 82 dual users, 50% reported that their child's healthcare provider discussed medicines to help them quit smoking, compared to 29% of cigarette-only smokers. Of these parents who reported that their child's healthcare provider discussed medicines to help them quit, 49% dual users and 39% cigarette-only smokers received a prescription for medicine to help them quit smoking. In addition, 33% of dual users reported that their child's healthcare provider discussed referral to a telephone quitline to help them quit smoking, compared to 19% of cigarette-only smokers. Of these parents who reported that their child's healthcare provider discussed referral to a telephone quitline, 50% dual users and 38% cigarette-only smokers received referral to a quitline.

The multivariable logistic regression model (Table 3) shows that parents were more likely to receive cessation treatment if they were dual users compared to cigarette-only smokers (OR 2.43, 95% CI 1.38, 4.29). Child's insurance status, parents' sex, education, and race were not associated with parental receipt of tobacco cessation treatment in the model.

Table 4 shows that in the usual-care control practices, 0 dual users reported discussing a prescription for NRT with their child's clinician in control practices compared to 41 (50.0%) in the intervention practices, and 2 (2.50%) dual users reported discussing enrollment in a quitline compared to 27 (33.3%) in the intervention practices. No dual users reported receiving either a NRT prescription or an enrollment in the quitline in the control arm compared to 19 (23.8%) dual users reporting receiving a NRT prescription and 15 (18.5%) dual users reporting enrollment in the tobacco quitline in the intervention practices.

## 4. Discussion

Data from this study shows that dual users who visited CEASE intervention practices were more likely to receive a cessation treatment than parents who smoke only cigarettes two years after intervention implementation after controlling for child's insurance, parents' sex, education, and race. The data also shows that dual users in the usual-care control practices reported not receiving any treatment.

As displayed in Table 1, almost 51% of smoking parents who received treatment have tried quitting smoking in the past three months and failed. Smokers who made prior quit attempts typically feel more motivated to quit than those parents who have not made a quit attempt [32]. Data in Table 2 shows that dual users are more likely to have made a quit attempt in the past 3 months and more likely to be planning to quit in the next 6 months compared to cigarette-only smokers. It is critical that these motivated parents get evidence-based smoking cessation treatments [33] to help them quit both smoking combustible tobacco and using e-cigarettes in order to protect their children from further tobacco smoke exposure and from exposure to potentially harmful byproducts produced by e-cigarettes.

CEASE practices were trained to ask all parents about tobacco use and to provide advice and assist every parent who smoked by discussing and providing evidence-based cessation treatment, but in this study, we found that clinicians in the intervention practices were more likely to screen dual users for tobacco use. As a result, clinicians in the intervention practices were more likely to discuss evidence-based treatment options with dual users than cigarette-only smokers. It is not clear why the providers screened or discussed treatment more often with dual users than cigarette-only smokers. It could be that the dual users may be more receptive to screening for tobacco use and discussing various treatments to help them quit smoking. Another reason for this may be that clinicians may perceive that e-cigarette use among cigarette smokers is motivated by a desire to quit smoking [34] and may think that these parents have already taken a step towards smoking cessation by using e-cigarettes. It has been previously reported that dual user parents may have higher rates of contemplating smoking cessation than parents who only smoke cigarettes [29]. Another possibility is that the clinicians may perceive that dual users and their children are at a higher risk of exposure to toxicants in tobacco smoke and e-cigarette aerosols [35] and may need help to quit. Additional research is needed to better understand the variation in smoking cessation screening and assistance delivery to dual users compared to cigarette-only smokers in the pediatric setting when a tobacco cessation intervention is implemented.

The rates of screening and discussing treatment options were higher in dual users compared to cigarette-only smokers, but the rates of acceptability of NRT prescriptions or quitline referral were also higher in dual users compared to cigarette-only smokers when any treatment was discussed with them. Of the parents with whom the medicine was discussed in the intervention practices, dual users were significantly more likely to accept prescriptions for NRT compared to cigarette-only smokers. Similarly, of the parents with whom quitline enrollment was discussed, dual user parents were significantly more likely to be enrolled in the quitline compared to cigarette-only smokers (Table 2). This data suggests that these dual user parents might be more receptive than cigarette-only smokers to cessation treatments when discussed and offered by their child's clinician. These dual user parents seem to be contemplating smoking cessation, and it has been previously reported that parents who are contemplating quitting in the near future are more likely to connect with the quitline and use the service [36]. This reinforces the important role of child healthcare providers in screening families for tobacco use and connecting them with the evidence-based treatments and resources to help them quit smoking [37].

When parental smokers quit smoking, they improve their own health [11], eliminate most of their children's exposure to tobacco smoke [1], and decrease the chances of their children starting smoking [38]. Despite clinical guidelines recommending that pediatric clinicians should address parental tobacco use and address children's tobacco smoke exposure [33, 39], the research showed that less than 1 percent of smokers received tobacco cessation treatment and no dual users in control practices received tobacco cessation treatment (Table 4). A US national parent survey data also showed low rates of recommending and prescribing cessation therapies [40]. These data suggest that significant opportunities exist to improve the rates of treating parents for tobacco use in the pediatric setting and reduce children's exposure to tobacco smoke. In light of persistent tobacco use by parents and the emerging epidemic of e-cigarettes, it is critical that all parents who use tobacco be offered evidence-based tobacco cessation treatments to help them quit smoking. Interventions like CEASE [31] have proven to be effective in creating a simple, innovative, and efficient way to screen families for tobacco use and get them treatment in the form of prescriptions for nicotine patches and gum, and referral to the state's free tobacco quitline.

### 4.1. Limitations

The sample size of dual users is relatively small so the results should be interpreted cautiously. In addition, results are based on cross-sectional exit-survey data and no causal inferences should be made for the observed associations. Finally, self-reported data are subject to recall and response bias. However, the administration of the survey, immediately following the clinical visit, decreased the likelihood of recall bias of the tobacco control services received at the office visit.

### 4.2. Public Health Implications

Dual users may have higher rates of contemplating smoking cessation than cigarette-only smokers and are more likely than cigarette-only smokers to receive tobacco cessation assistance after having discussions about NRT and/or the tobacco quitline with clinicians. An increased uptake of tobacco cessation treatments among dual users reinforces the importance of discussing treatment options with this group, while also recognizing that cigarette-only smokers may require additional intervention to increase the acceptance rate of cessation assistance.

## Figures and Tables

**Table 1:** Characteristics of currently smoking parents who received treatment^∗^ to help them quit vs. who did not receive treatment, 2 years post-CEASE implementation in the intervention arm (*N* = 802).

Characteristic	Received treatment *N* = 113 *n* (%)	Did not receive treatment *N* = 689 *n* (%)	*p* value
Parent age			0.544
18-24	15 (13.27)	129 (18.72)	
25-44	86 (76.11)	490 (71.12)	
≥45	12 (10.62)	70 (10.16)	
Relationship to the child			0.197
Father	25 (22.12)	142 (20.61)	
Mother	84 (74.34)	489 (70.97)	
Other	4 (3.54)	58 (8.42)	
Hispanic	3 (2.65)	25 (3.63)	0.601
Race			0.057^∗∗^
Non-Hispanic Black or African American	8 (7.08)	27 (3.92)	
Other or >1 race	3 (2.65)	65 (9.43)	
Non-Hispanic White	103 (91.15)	641 (93.03)	
Education			0.030^∗∗^
<High school	13 (11.50)	93 (13.50)	
High school graduate	57 (50.44)	297 (43.11)	
Some college	42 (37.17)	242 (35.12)	
College graduate	1 (0.88)	57 (8.27)	
# cigarettes/day			0.257
1-10 cigarettes/day	46 (40.71)	320 (46.44)	
≥11 cigarettes/day	67 (59.29)	369 (53.56)	
Plan to quit			
Next 6 months	93 (87.74)	478 (75.39)	0.005^∗∗^
Next 30 days	67 (79.76)	271 (64.22)	0.006^∗∗^
Quit attempt in the last 3 months			
Yes	57 (50.89)	332 (48.40)	0.624
Daily smoker	97 (85.84)	565 (82.36)	0.363
Youngest child seen age			0.398
<1 year	30 (26.79)	240 (34.83)	
1-4 years	24 (21.43)	141 (20.46)	
5-9 years	28 (25.00)	150 (21.77)	
≥10 years	30 (26.79)	158 (22.93)	
Child's insurance coverage			0.377
Medicaid	90 (80.36)	527 (76.71)	
Self-pay	4 (3.57)	14 (2.04)	
Private insurance/HMO	17 (15.18)	143 (20.82)	
Dual user	22 (19.47)	59 (8.56)	0.000^∗∗^
Asked about smoking status	104 (92.04)	281 (40.78)	0.000^∗∗^

^∗^Parents were considered to have received tobacco cessation treatment if they answered “yes” to receiving a prescription for medicine to help them quit and/or being enrolled in the telephone quitline. ^∗∗^*p* value < 0.05.

**Table 2:** Intention to quit and smoking cessation assistance delivery among dual user parents vs. cigarette-only smokers 2 years post-CEASE implementation in the intervention arm (*N* = 804).

	Dual user (*N* = 82) *n* (%)	Cigarette only smoker (*N* = 722) *N* (%)	*p* value
Parent age			0.630
18-24	15 (18.3)	129 (17.9)	
25-44	62 (75.6)	516 (71.5)	
≥45	5 (6.1)	77 (10.67)	
Relationship to the child			0.012
Father	26 (31.7)	142 (19.7)	
Mother	54 (65.9)	520 (72.0)	
Other	2 (2.4)	60 (8.3)	
Hispanic	2 (2.4)	26 (3.6)	0.586
Race			
Non-Hispanic Black or African American	2 (2.4)	33 (4.6)	0.370
Other or>1 race	4 (4.9)	21 (2.9)	0.330
Non-Hispanic White	77 (93.9)	669 (92.7)	0.680
Education			0.538
<high school	15 (18.3)	91 (12.6)	
High school graduate	33 (40.2)	322 (44.6)	
Some college	28 (34.2)	257 (35.6)	
College graduate	6 (7.3)	52 (7.20)	
# cigarettes/day			0.139
1-10 cigarettes/day	31 (37.8)	335 (46.4)	
≥11 cigarettes/day	51 (62.2)	387 (53.6)	
Plan to quit			
Next 6 months	69 (88.5)	504 (75.9)	0.012^∗^
Next 30 days	47 (75.8)	293 (65.7)	0.113
Quit attempt in the last 3 months			
Yes	57 (69.5)	332 (46.2)	0.000^∗^
	48 (58.54)	339 (46.95)	0.047^∗^
Advice to quit	43 (52.44)	270 (37.40)	0.008^∗^
Discuss medicine to quit	41 (50.00)	209 (29.03)	0.000^∗^
Received prescription	19 (23.75)	83 (11.53)	0.002^∗^
Prescription acceptance ratio when offered by the pediatric staff (received prescription/discuss medicine)	19 (48.7)	81 (38.9)	0.254
Discuss quitline	27 (33.33)	134 (18.61)	0.002^∗^
Referred to the quitline	15 (18.52)	51 (7.08)	0.000^∗^
Quitline referral ratio when offered by the pediatric staff (referred to the quitline/discuss quitline)	13 (50.0)	50 (37.6)	0.237

*p* values < 0.05.

**Table 3:** Multivariable logistic regression model predicting delivery of smoking cessation treatment (receiving prescriptions and/or enrollment in quitline) 2 years post-CEASE implementation in the intervention arm (*N* = 734).

Variable	OR (95% CI)
Dual use of cigarettes and e-cigarettes	
Yes	2.43 (1.38, 4.29)^∗^
No (cigarette-only smoker)	1.0^a^
Insurance status	
Medicaid	1.37 (0.92, 2.06)
Private insurance or self-pay	1.0^a^
Relationship to the child	
Mother	1.08 (0.72, 1.62)
Father	1.0^a^
Race	
White	0.72 (0.47, 1.11)
Non-White	1.0^a^
Education	
Less than high school	0.69 (0.40, 1.20)
Completed high school or college	1.0^a^

^a^Indicates reference group, ^∗^*p* < 0.05.

**Table 4:** Smoking cessation assistance delivery among dual user parents vs. cigarette-only smokers 2 years post-CEASE implementation in the control vs. intervention arms (*N* = 1531).

Characteristic	Current cigarette-only smokers			Dual users		
	Control *N* = 646 *N* (%)	Intervention *N* = 722 *N* (%)	*p* value	Control *N* = 81 *N* (%)	Intervention *N* = 82 *N* (%)	*p* value
Discuss medicine to quit	16 (2.5)	209 (29.0)	<.001	0 (0)	41 (50.0)	<.001
Discuss enrollment in state quitline	13 (2.0)	134 (18.6)	<.001	2 (2.5)	27 (33.3)	<.001
Prescribe NRT	2 (0.3)	83 (11.5)	<.001	0 (0)	19 (23.8)	<.001
Enroll in quitline	0 (0)	51 (7.1)	<.001	0 (0)	15 (18.5)	<.001

## Data Availability

A deidentified dataset and a data dictionary with information describing the variables will be shared with other researchers on request. All requests should be emailed to Jonathan P. Winickoff, MD, MPH at jwinickoff@mgh.harvard.edu.
